# Combined Administration of Streptozotocin and Sucrose Accelerates the Appearance of Type 2 Diabetes Symptoms in Rats

**DOI:** 10.1155/2019/3791061

**Published:** 2019-07-04

**Authors:** Martha Isela Barragán-Bonilla, Juan Miguel Mendoza-Bello, Penélope Aguilera, Isela Parra-Rojas, Berenice Illades-Aguiar, Mónica Ramírez, Mónica Espinoza-Rojo

**Affiliations:** ^1^Laboratorio de Biología Molecular y Genómica de la Facultad de Ciencias Químico-Biológicas, Universidad Autónoma de Guerrero, Av. Lázaro Cárdenas S/N, Ciudad Universitaria, Chilpancingo de los Bravo, Gro. 39090, Mexico; ^2^Laboratorio de Patología Vascular Cerebral, Instituto Nacional de Neurología y Neurocirugía “Manuel Velasco Suárez”, Av. Insurgentes Sur 3877, Mexico City 14269, Mexico; ^3^Laboratorio de Obesidad y Diabetes de la Facultad de Ciencias Químico-Biológicas, Universidad Autónoma de Guerrero, Av. Lázaro Cárdenas S/N Ciudad Universitaria, Chilpancingo de los Bravo, Gro. 39090, Mexico; ^4^Laboratorio de Biomedicina Molecular de la Facultad de Ciencias Químico-Biológicas, Universidad Autónoma de Guerrero, Av. Lázaro Cárdenas S/N, Ciudad Universitaria, Chilpancingo de los Bravo, Gro. 39090, Mexico; ^5^CONACYT-Universidad Autónoma de Guerrero, Av. Javier Méndez Aponte No. 1, Fracc. Servidor Agrario, Chilpancingo de los Bravo, Gro. 39070, Mexico

## Abstract

Type 2 diabetes is a disease with a high global prevalence, characterized by chronic hyperglycemia, insulin resistance, polyphagia, polydipsia, polyuria, and changes in body weight. Animal models have been very useful for the study of this disease and to search for new therapeutic targets that delay, attenuate, or avoid diabetic complications. The purpose of this work was to establish a model of type 2 diabetes and exhibit the majority of the characteristics of the disease. Two-day-old male and female Wistar rats were treated once with streptozotocin (70 or 90 mg/kg body weight). After weaning, they were given a sucrose-sweetened beverage (SSB; sucrose at 10 or 30%) during 7 or 11 weeks; their body weight and food intake were measured daily. With the rats at 14 weeks of age, we determined the following: (a) fasting blood glucose, (b) oral glucose tolerance, and (c) insulin tolerance. We found that the supplementation of sucrose at 10% for 7 weeks in male rats which had previously been given streptozotocin (70 mg/kg) at neonatal stage leads to the appearance of the signs and symptoms of the characteristic of type 2 diabetes in adulthood.

## 1. Introduction

Type 2 diabetes is a multifactorial and degenerative disease characterized by chronic hyperglycemia and insulin resistance, in addition to symptoms such as polyuria, polydipsia, and polyphagia. Due to the magnitude of this problem worldwide [1, 2], the implementation of representative animal models to study this disease is relevant in order to improve treatments and to attenuate or avoid diabetic complications [3, 4].

Despite that there are many animal models that allow the study of some aspects of this disease, there is none, to our knowledge, that shows the majority of the features of type 2 diabetes [3, 4]. In some models, hyperglycemia is induced in adult rats by the administration of streptozotocin (STZ); the mechanism of action is through the selective destruction of pancreatic *β* cells by oxidative stress. Consequently, this decreases insulin in circulation, increases the blood glucose level, and brings on the presence of severe symptoms [5–8].

STZ has also been used in neonatal rats (nSTZ model) to generate a similar model of type 2 diabetes. This animal model generates partial damage to pancreatic *β* cells, due to regeneration of these cells at neonatal age, which favors the presence of glucose intolerance, insulin resistance, and insulin deficiency in early adulthood [9–13]. In the literature, we find widely employed protocols to induce diabetes through the administration of STZ; the latter has been used at mild-to-high doses (range, 70-150 mg/kg) at different postnatal ages (2-5 days of age). This diversity of protocols reflects a low efficiency for their use as study models of the disease. In fact, it has been reported that there is no reproducibility in the generation of alterations associated with type 2 diabetes [10–23]. Therefore, it is important to establish a model of type 2 diabetes that manifests the majority of the characteristics of the disease.

On the other hand, it has been reported that high-fat and high-carbohydrate diets, or supplementation with sweetened beverages for a long time period, leads to the generation of a model of insulin resistance and obesity. However, there is a wide variety of protocols, in addition to the fact that special solid diets are expensive and supplementation with sweetened beverages is often used for excessively long time periods [24–27].

In this context, the purpose of this study was to evaluate a different model to study type 2 diabetes by using STZ—in 2 days old rats—in combination with sweetened beverages (sucrose-sweetened beverage (SSB)) and to determinate if that combination accelerates the establishment of type 2 diabetes symptoms in rats.

## 2. Materials and Methods

### 2.1. Animals

Two days old male (*n* = 70) and female (*n* = 68) Wistar rats were used, which were donated by the National Institute of Neurology and Neurosurgery “Manuel Velasco Suárez” (INNN), Mexico City. During the study, the rats were maintained under constant humidity (50-60%) and temperature (21-25°C), with light and dark cycles of 12 h/12 h and free access to food (Harlan's standard commercial diet No. 2018S Teklad Global 18% Protein Rodent Diet) and water. They were treated with the greatest possible care to avoid pain and suffering, under the conditions indicated in Mexican NOM-062-ZOO-1999 (Technical Specifications for the Production, Care and Use of Laboratory Animals) and the rules established by the Ethics Commission of the INNN, Protocol 32/17 with document No. CICUAL/SO/VI/22617/028/2017.

### 2.2. Experimental Protocols

**Figure 1:**
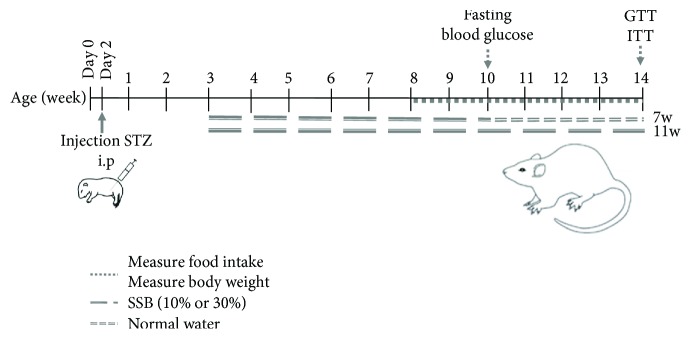
Experimental scheme of diabetes induction in Wistar rats. Two-day-old male and female rats were injected with STZ (70 or 90 mg/kg) intraperitoneally (i.p.). The rats were immediately returned to their mothers and weaned at 21 days of age. In addition, rat groups with and without 70 mg of STZ/kg body weight were supplemented with SSB (standard commercial sucrose) at 10% or 30% for either 7 or 11 weeks after weaning. The Control group was included that was not exposed to STZ nor to SSB. Body weight and food intake were monitored daily for 6 weeks (when the rats were 8-14 weeks of age), and fasting blood glucose was measured at weeks 10 and 14 of age. Afterward, the insulin tolerance test (ITT) and glucose tolerance test (GTT) were performed at week 14.

We included two experimental protocols to induce alterations in glucose homeostasis: experimental protocol A: a single dose of STZ (Cat. number S0130; Sigma-Aldrich, St. Louis, MO, USA), and experimental protocol B: a single dose of STZ with SSB:

- Wistar rats (two days old) were fasted for 8 h, while being separated from their mothers; then, they received either vehicle (25 *μ*l of buffer 0.1 M sodium citrate pH 4.5) or a single dose of STZ (70 or 90 mg/kg body weight) via intraperitoneal (i.p.) injection. For each experimental group, the rats were randomly divided into three groups (Control, Stz70, and Stz90). The rats were immediately returned to their mothers and weaned at 21 days of age (Figure 1)
- High-sugar diets are determinant for establishing insulin resistance. For that reason, different groups of rats were formed with and without the neonatal administration of 70 mg of STZ. After weaning, the rats were supplemented with 10% or 30% SSB during 7 or 11 weeks (standard commercial sucrose according to the specifications of NOM051-SCFI/SSA1/2010) with the objective of determining the adequate concentration of sucrose and the time for it to be administered. The Control group received only vehicle of STZ (Figure 1)

Rats of both experimental protocols were organized randomly into 11 study groups as follows: (a) Control group, rats not exposed to either STZ or SSB (males *n* = 9, females *n* = 9); (b) Stz70 group, animals treated with a single dose of 70 mg of STZ (males *n* = 8, females *n* = 9); (c) Stz90 group, animals with a single dose of 90 mg of STZ (males *n* = 4, females *n* = 2); (d) C+10% 7w group, control animals treated with 10% SSB during 7 weeks (males *n* = 6, females *n* = 6); (e) C+10% 11w group, control animals treated with 10% SSB during 11 weeks (males *n* = 6, females *n* = 6); (f) C+30% 7w group, control animals treated with 30% SSB during 7 weeks (males *n* = 6, females *n* = 6); (g) C+30% 11w group, control animals treated with 30% SSB during 11 weeks (males *n* = 6, females *n* = 7); (h) Stz70+10% 7w group, animals treated with a single dose of 70 mg of STZ and 10% SSB during 7 weeks (males *n* = 7, females *n* = 6); (i) Stz70+10% 11w group, animals treated with a single dose of 70 mg of STZ and 10% SSB during 11 weeks (males *n* = 6, females *n* = 5); (j) Stz70+30% 7w group, animals treated with a single dose of 70 mg of STZ and 30% SSB during 7 weeks (males *n* = 6, females *n* = 6); and (k) Stz70+30% 11w group, animals treated with a single dose of 70 mg of STZ and 30% SSB during 11 weeks (males *n* = 6, females *n* = 6).

After weaning, the rats were placed in cages with four rats per cage. At 5 weeks of age, the rats were sexed and housed in cages with two animals each. Afterward, at 6 weeks of age, the rats were separated into individual boxes and were handled periodically to habituate them to management during the monitoring of the parameters, allowing us to measure parameters that would permit the complete diagnosis of diabetes.

### 2.3. Evaluation of the Diabetic Condition

At 8 weeks of age, body weight and food intake were measured daily for 6 weeks to determine changes in body weight and polyphagia. At weeks 10 and 14, the fasting blood glucose level was measured to determine whether the animals had hyperglycemia. Additionally, at week 14, an oral glucose tolerance test (GTT) and an insulin tolerance test (ITT) were performed to assess alterations in glucose homeostasis and insulin sensitivity, respectively (Figure 1).

### 2.4. Measurement of Body Weight and Food Intake

Body weight was measured on an analytical scale with a basket added for this purpose. To measure food intake daily, pellets of food were previously weighed and placed in the cages at 8:00 am; after 24 h, pellets were removed and weighed again. The result was adjusted to the body weight (g of food/100 g body weight per day).

### 2.5. Measurement of Fasting Blood Glucose Levels, GTT, and ITT

The blood glucose concentration was evaluated with an 8 h fast. The OneTouch® UltraTM diagnostic kit was used, following the manufacturer's instructions. For the GTT, the animals were given glucose (2 g/kg body weight) orally [15], and for the ITT, they were injected with insulin (0.5 IU/kg body weight) i.p. Then, glucose concentrations were monitored at 30, 60, and 120 min after the administration of glucose or insulin. Rats with fasting glucose ≥ 200 mg/dl were considered hyperglycemic, and the GTT was not performed on these animals.

The ITT was employed to determinate insulin resistance, which is quite a simple, fast, reproducible, and inexpensive assay, and previous research has validated and compared it with other tests that have a greater acceptance to determine insulin resistance, like mathematical model HOMA-IR and euglycemic hyperinsulinemic clamp, and have shown a positive correlation [28, 29].

### 2.6. Data Analysis

Data are expressed as the median and interquartile range (IQR, p25-p75). Normality of data was evaluated by the Shapiro-Wilk test. Data of the GTT and ITT are represented in percentages, considering the initial glucose value as 100%. The area under the curve (AUC) was calculated using the trapezoidal rule. The variation between more than two groups was measured by the Kruskal-Wallis test followed by the Dunn Multiple Comparison Test. The analysis was performed using the GraphPad Prism ver. 5.0 statistical software. The statistical significance was considered with a value of *p* < 0.05.

## 3. Results

### 3.1. Effects of the Administration of STZ at Neonatal Age on Survival and Glucose Tolerance

We injected 90 mg of STZ into 42 male and 38 female rats and found that this dose induced high mortality (90.5 and 94.7%, respectively) during the first days after STZ administration. In contrast, 39 male and 37 female rats were injected with 70 mg of STZ, and a high percentage of animals of this group survived, with 84.6 and 86.5%, respectively (data not shown).

The rats from the Stz90 group, which survived and reached adulthood (14 weeks-of-age), had a fasting blood glucose level close to (female rats) or greater than 200 mg/dl (male rats) (both *p* < 0.05 vs. the Control groups). In contrast, animals from the Stz70 group showed a fasting glucose level near that of the Control group (Table 1).

To confirm alterations in glucose tolerance in rats that received neonatal STZ and had fasting glycemia of <200 mg/dl (i.e., male and female rats from the Stz70 group and females from the Stz90 group), the GTT was performed and the AUC was calculated. After 30, 60, and 120 min of the administration of glucose via oral, the blood glucose level in both groups of male and female rats was higher than that of the Control group (*p* < 0.001). In addition, the AUC of both groups formed by males (Stz70: AUC = 796.2) and females (Stz70: AUC = 822.4; Stz90: AUC = 1,165) was higher than the AUC of the Control group (*p* < 0.05; AUC of male control: 505.4, AUC of female control: 547.5; Table 2). The data showed the presence of glucose intolerance in these groups.

### 3.2. Effects of the Administration of STZ at Neonatal Age on Insulin Sensitivity

An ITT was performed to determine whether the administration of STZ affects the body's efficiency in response to exogenous insulin. For this purpose, insulin was administered i.p., and we measured blood glucose levels after 30, 60, and 120 min. We found that there was a diminished response to insulin only after 30 min in male rats from the Stz70 group and in male and female rats from the Stz90 group in comparison with the Control group (glucose level approximately 8, 19, and 50% higher than that of the Control group at 30 min, respectively; *p* < 0.05). These data indicate a low response to insulin. However, according to the AUC of different groups comprising males and females, it did not exhibit any change in comparison with the Control group; therefore, these data, taken together, demonstrate a mild resistance to insulin (Table 2).

### 3.3. Administration of 90 mg of STZ at Neonatal Age Altered Body Weight and Induced Polyphagia

To identify the presence of the symptoms of diabetes as changes in body weight and polyphagia, we measured body weight and food intake daily for 6 weeks (8-14 weeks of age). During that period, male and female rats from the Control group had a body weight gain of 152 and 78 g, respectively. Male and female rats from the Stz70 group demonstrated a similar body weight gain to that of the Control group (150.5 and 68.1 g, respectively). In contrast, in the Stz90 groups formed by male and female rats, a lower body weight gain was found (a difference of approximately 30–50 g vs. the Control group; *p* < 0.05) (Table 5).

In relation to food intake, male and female rats from the Control group had a food intake of 6.9 and 7.7 g/100 g of body weight, respectively. We observed that there was a significant increase in daily food intake in males and females of the Stz90 group (12.2 and 13.2 g/100 g of body weight, respectively, *p* < 0.0001, compared with the Control group). In contrast, male and female rats from the Stz70 group did not show any changes in food intake in comparison with the Control group (Table 5).

### 3.4. Effects of the Supplementation of SSB on Fasting Blood Glucose and Glucose Tolerance in Rats with Previous Inoculation of STZ at Neonatal Age

Considering that the administration of 70 mg of STZ promotes alterations in glucose homeostasis without affecting its concentration in terms of fasting state, body weight, or food intake, this dose was selected and combined with SSB (10% or 30% sucrose for 7 or 11 weeks; Figure 1).

Control rats that were given SSB at different concentrations and duration times (C+10% 7w, C+10% 11w, C+30% 7w, and C+30% 11w) showed their fasting blood glucose level in a range similar to that of the Control group regardless of sex. In contrast, rats with 70 mg of STZ and SSB exhibited an increase in the blood glucose level in all groups of both sexes (Stz70+10% 7w, Stz70+10% 11w, Stz70+30% 7w, and Stz70+30% 11w; *p* < 0.05 vs. Control). However, only the Stz70+10% 7w group revealed an increase in glucose of >200 mg/dl in male rats (Table 3).

The GTT was carried out to evaluate the effect of SSB on glucose tolerance in rats with a previous administration of neonatal STZ that had a fasting glycemia of <200 mg/dl. The data from the AUC showed that 30% SSB for 7 weeks generated glucose intolerance in male rats (*p* < 0.05; AUC = C+30% 7w: 650.4 vs. Control: 505.4). In contrast, groups made up of females did not show changes in the blood glucose level in response to oral glucose load with respect to the Control group (Table 4).

On the other hand, with supplementation of 10% or 30% SSB at either 7 or 11 weeks after weaning, male rats treated with neonatal STZ showed higher glucose intolerance (AUC = Stz70+10% 7w: 722.1, Stz70+10% 11w: 737.8, Stz70+30% 7w: 984.6, and Stz+30% 11w: 876.8) than control animals (AUC = 505.4; *p* < 0.05) (Table 4). In the same way, female rats with neonatal STZ and supplemented with SSB during different periods, as expected, presented higher glucose intolerance than that of the Control group (AUC = Stz70+10% 7w: 630, Stz70+10% 11w: 708.3, Stz70+30% 7w: 859, and Stz70+30% 11w: 1.081; *p* < 0.05 vs. Control: 547.5) (Table 4).

### 3.5. Effects of Supplementation of SSB on Insulin Resistance in Rats with Previous Inoculation of STZ at Neonatal Age

In relation to the evaluation of insulin resistance through the ITT, we found that, in male rats, insulin sensitivity was significantly diminished after 30 min postinsulin in male rats from the C+10% 7w, C+30% 7w, and Stz70+10% 7w groups (glucose level approximately 30% higher than that of the Control group after 30 min, respectively; *p* < 0.05). This data indicated a low response to insulin in these groups. However, data from AUC showed a lower response only in the C+10% 11w and C+30% 7w groups (AUC = 341.4 and 375.5, respectively, vs. 288.7 of the Control group; *p* < 0.05, Table 4).

In contrast, female rats with neonatal STZ plus 10 or 30% SSB administered for 7 weeks revealed an increase in insulin sensitivity (AUC = 184.4 and 199.6, respectively, vs. 315.9 in the Control group; *p* < 0.05) (Table 4). The remainder of the groups formed by female rats showed a similar response to insulin to that of the Control group (Table 4).

### 3.6. Effects of Supplementation of SSB on Body Weight and Polyphagia in Rats with Previous Inoculation of STZ at Neonatal Age

Supplementation with 10 or 30% SSB either for 7 or for 11 weeks in males without neonatal STZ promoted a higher body weight gain of approximately 30 g in comparison with the Control group (*p* < 0.05 vs. Control). On the other hand, in males with STZ plus 10% SSB during 7 or 11 weeks, a lower body weight gain was favored (a difference was found of 50-60 g vs. Control; *p* < 0.05). In groups made up of females, there were no changes in body weight gain (Table 5).

Regarding food intake, we observed that there was an increase in daily food intake in males of the Stz70+30% 7w and Stz+10% 7w groups (9.3 and 12.3/100 g body weight, respectively; *p* < 0.05 vs. 6.9/100 g body weight of the Control group). In contrast, the groups of male and female rats with and without neonatal STZ that were supplemented with 10 or 30% SSB permanently (11 weeks) exhibited a decrease in daily food intake compared with the Control group (*p* < 0.05) (Table 5).

## 4. Discussion

In our research, we found that 90 mg of STZ induced the majority of the characteristics of diabetes (hyperglycemia, low body weight gain, and polyphagia) in both males and females; in fact, this dose is widely used [9, 15, 25, 30, 31]. However, its implementation in this research is difficult as a study model due to that it promotes high mortality in animals.

On the other hand, we demonstrated that 70 mg of STZ only generates glucose intolerance, without affecting body weight gain or food intake. Its lower effect could be due to the damage generated to pancreatic *β* cells dependent on the concentration of STZ [9, 12]. It will be determinant to evaluate the damage level in pancreatic *β* cells in both models.

As part of the establishment of a type 2 diabetes model, a single dose of 70 mg of STZ was selected to be employed in combination with SSB (10 or 30%) during either 7 or 11 weeks. This is due to the fact that the injection of this dose only induces glucose intolerance, without affecting body weight or food intake. Additionally, we included control groups with SSB (10 or 30%) during either 7 or 11 weeks in order to discard those alterations in glucose homeostasis generated solely by SSB.

In our study, we found that supplementation with SSB in male and female rats without the administration of STZ did not affect the fasting blood glucose levels neither the normal response to the administration of glucose in GTT. Despite the fact that previous reports have demonstrated that only SSB or a high-carbohydrate diet during a chronic period leads to impairment to glucose homeostasis and obesity [24–27] in our study, supplementation with SSB was not a sufficient factor to generate alterations in glucose homeostasis, because SSB was given for a shorter time (7 or 11 weeks) in comparison with other studies. It is probable that the organism can compensate for the high sucrose intake through beverages by promoting greater synthesis and the release of insulin from pancreatic *β* cells. Therefore, it is possible that hyperinsulinemia is responsible for avoiding an abnormal response in the GTT. Actually, it has been described that an increased metabolic demand induces an increase of *β* cell's function or *β* cell mass expansion to compensate for the condition. *β* cell mass expansion can be due to the self-duplication of preexisting *β* cells or to the transdifferentiation of pancreatic *α* cells [32, 33]. In future studies, it will be necessary to measure the concentration of insulin and to identify the *β* cell mass expansion that would permit us to explain this finding with precision.

In the case of rats with STZ plus SSB at different concentrations and times, we found that SSB stimulated an increase in baseline blood glucose concentration and a low response to glucose administered in the GTT. The mechanism of action of STZ administered at the neonatal age is to induce partial damage to the pancreatic *β* cells, thus generating alterations in glucose homeostasis and the symptomatology of diabetes in a gradual manner in adulthood. This means that pancreatic *β* cells will still be able to produce insulin in response to high blood glucose levels (e.g., after eating food) [9–13]. In this manner, by constantly stimulating the production of insulin through a moderate intake of sucrose in a beverage, it could promote the development of greater damage to the pancreatic *β* cells. Due to partial damage of pancreatic *β* cells at the neonatal age, the capacity of expansion of *β* cells can be limited in these rats, resulting in a failure to compensate for increased metabolic needs [34]. This event, therefore, could promote the establishment of fasting hyperglycemia in rats with STZ and SSB.

Interestingly, fasting hyperglycemia is more pronounced in groups with STZ plus SSB at low concentrations and short time periods (i.e., 10% of sucrose for 7 weeks). This effect could be explained as due to an effect of the compensation of the organism, meaning that, when rats are supplemented with a higher concentration of sucrose (30%) and at a prolonged time (11 weeks), there is an adaptation of the functional *β* cell mass to promote greater production of insulin by the pancreatic *β* cells, preventing with this the early establishment of the symptoms of diabetes [9–13]. Thus, it is thought that with low concentrations of sucrose during a short time period, the adaptation stage of the organism does not occur; thus, the damage generated in the *β* cells becomes more evident in earlier stages with hyperglycemia and the symptoms of diabetes [34].

In relation to insulin resistance, a key characteristic of type 2 diabetes mellitus, previous reports have shown that the administration of neonatal STZ induces insulin resistance [14, 17, 18]. In this study, the injection of 70 mg of STZ in male and female rats or 90 mg of STZ in female rats leads to a mild response to exogenous insulin. Likewise, male control rats that were given 10 or 30% SSB for 7 weeks and male rats that received 10% SSB during the same period with a previous injection of STZ showed mild insulin resistance. Insulin intolerance appears to be greater than in rats with only an injection of STZ. This could be due to the fact that in addition to neonatal STZ, the sucrose has been reported as a factor that promotes insulin resistance [24, 27]. However, it is necessary to determine whether this low insulin response is due to the poor density of insulin receptors on tissues, defects in its signaling pathway, or the oxidative damage of exogenous insulin [35, 36].

In contrast, in groups formed by females, supplementation with SSB do not change insulin tolerance. This condition could be due to the fact that the animals utilized in this study were young adults and, at this stage, the high estrogen levels in females act as a protective factor against the development of diabetes [37, 38], thus avoiding the appearance of hyperglycemia and the rest of the characteristic diabetic symptoms in females.

Otherwise, we observed that a prolonged intake of SSB (11 weeks) led to a decrease in food intake in all groups of both sexes; this can be because chronic exposure to a moderate or high concentration of sucrose might promote satiety. It is probable that the constant consumption of sucrose through drinking stimulates hypothalamic signals of satiety, which would explain the low consumption of solid food. Although there is no clinical evidence to support this assumption, it is possible that the free glucose obtained by the degradation of sucrose is responsible for inducing satiety. Glucose in high concentrations may promote satiety signals in the hypothalamus [39].

Rats that were injected with STZ and supplemented with 10% SSB for a moderate period (7 weeks) exhibited polyphagia. This can be explained that, by means of the compensation mechanism described previously, the capacity of expansion of *β* cells can be limited in these rats due to the partial damage of pancreatic *β* cells that was generated at neonatal age. Therefore, this results in a failure to compensate increased metabolic needs [34].

In addition, we found that the consumption of SSB gave rise to an increase in body weight gain in male rats, but not in female rats. Previous studies have reported that high-carbohydrate diets lead to body weight gain [26, 40]. This is due to the fact that the excess of carbohydrates in the diet is stored in adipose tissue in the form of triglycerides, leading to an increase in the body mass of the individual. To support this, some studies have demonstrated an association between sucrose intake and triglycerides [41].

Contrariwise, male and female rats that were injected with 90 mg of STZ and male rats that received 10% SSB for 7 or 11 weeks in addition to the STZ had a lower body weight gain. Considering that these rats had hyperglycemia, the low body weight gain in diabetes is explained as due to the inability of the cells to use glucose as the main source of energy. Thus, it degrades fatty acids and proteins to maintain the vital functions of the organism [5–7].

In summary, in this research, the following two different and important models were generated in Wistar rats that might be useful in research on diabetes: (a) male and female rats with neonatal STZ (70 mg/kg) have mild insulin resistance and glucose intolerance, and (b) male rats with neonatal STZ plus 10% SSB for 7 weeks developed hyperglycemia, mild insulin resistance, low body weight gain, and polyphagia; therefore, we considered the latter model as a model that is more similar to type 2 diabetes. In addition, we can reproduce a diabetes model with 90 mg/kg of STZ; however, we emphasize that its use in research can be difficult because this dose induces high mortality. The use of each of the two models will depend on the purpose of the study.

In general, these models have the advantage of being inexpensive due to the fact that the STZ is administered at neonatal age; therefore, the amount of this drug employed is minimal compared to when it is used in adult age rats. On the other hand, the use of sweetened beverages with commercial sugar for a short time is cheaper than the use of special solid food (high-fat and high-carbohydrate diets).

## Figures and Tables

**Table 1:** Fasting blood glucose (mg/dl) of rats at 10 and 14 weeks of age of experimental protocol A. The data shown are of the Control (males *n* = 9, *n* females *n* = 9), Stz70 (males *n* = 8, females *n* = 9), and Stz90 (males *n* = 4, females *n* = 2) groups. Values are expressed as the median and interquartile range (IQR) (p25-p75). The Kruskal-Wallis test was used for testing the statistical significance between groups of data, in addition to a posthoc Dunn Multiple Comparison Test (Control, Stz70, and Stz90). The asterisk denotes that the data are significantly different from the Control group (^∗^*p* < 0.05, ^∗∗^*p* < 0.01, and ^∗∗∗^*p* < 0.001), and the plus sign denotes that the data are significantly different from the Stz70 group (^+^*p* < 0.05, ^++^*p* < 0.01, and ^+++^*p* < 0.001).

	Male		Female	
	10^th^ week	14^th^ week	10^th^ week	14^th^ week
Control	97 (91.3-116.8)	106.5 (94.5-119.5)	93 (92-118)	96.5 (89.5-105)
Stz70	129.5 (120.3-144.8)	131 (107-248)	131.5 (106.5-153.5)	138.5∗∗ (113-206)
Stz90	164.5 (119-224)	**278.5**∗∗^**+**^ (228.3-395.5)	190.5∗∗ (151.3-276.3)	**190**∗∗ (174.3-247)

**Table 2:** Glucose tolerance test (GTT) and insulin tolerance test (ITT) of rats of experimental protocol A. The data shown are of the Control (males *n* = 9, females *n* = 9), Stz70 (males *n* = 8, females *n* = 9), and Stz90 (males *n* = 4, females *n* = 2) groups. Data are represented in percentages, considering the initial glucose value as 100%. Values (except initial glucose) are expressed as the median and interquartile range (IQR) (p25-p75). For the GTT, rats were given glucose (2 g/kg body weight) orally, and for the ITT, they were injected with insulin (0.5 IU/kg body weight) i.p. Blood glucose level was monitored at 30, 60, and 120 min after the administration of glucose or insulin. The area under the curve (AUC) was calculated using the trapezoidal rule in GraphPad Prism. Variation between more than two groups was measured by the Kruskal-Wallis test followed by Dunn Multiple Comparison Test. The asterisk denotes that data are significantly different from the Control group (^∗^*p* < 0.05, ^∗∗^*p* < 0.01, and ^∗∗∗^*p* < 0.001), and the plus sign denotes that the data are significantly different from the Stz70 group (^+^*p* < 0.05, ^++^*p* < 0.01, and ^+++^*p* < 0.001).

		GTT					ITT				
		0 min	30 min	60 min	120 min	AUC	0 min	30 min	60 min	120 min	AUC
Male	Control	100	147.5 (116.1-180.6)	133.1 (129.7-156)	114.3 (97.46-133)	505.4 (459.4-575.2)	100	55.4 (51.08-63.73)	68.8 (48.96-74.9)	80 (58.6-89.3)	288.7 (247.2-303.2)
	Stz70	100	236∗∗ (190.9-279.4)	216.7∗∗ (195.5-247.7)	184.1∗∗ (151.3-192.2)	796.2∗∗∗ (689.5-860.8)	100	**63.6**∗ (61.8-89.8)	58 (37.1-66.7)	76.6 (48.2-98.8)	279 (213.8-362.4)
	Stz90	—	—	—	—	—	100	50 (23.3-95.2)	40.4 (23.6-77.9)	38.4 (32.9-75.6)	179.8 (142.9-318.7)

Female	Control	100	174.7 (143.9-196.4)	149 (125.3-160.5)	113.3 (101.5-149.7)	547.5 (484.1-632.7)	100	54.4 (52.3-60.2)	64.2 (48.5-75)	87.5 (72.7-101.1)	315.9 (258.2-352)
	Stz70	100	253.7∗∗ (205.7-286.5)	243.4∗∗∗ (227.8-277.2)	158.3 (118.4-220.6)	822.4∗∗ (803.7-926.2)	100	**73.3**∗ (56.9-123.5)	75.2 (49.6-121.7)	110.5 (79.8-142.9)	318.8 (230.6-342.9)
	Stz90	100	364.7∗∗^++^ (332-397.4)	320∗∗^++^ (319.5-320.5)	269.7∗∗^++^ (230.8-308.6)	1165∗∗^++^ (1092-1237)	100	**104.5**∗ (77.4-131.6)	94.4 (43.4-145.8)	71.6 (21.2-122.1)	368 (213.6-522.4)

**Table 3:** Fasting blood glucose (mg/dl) in rats 10 and 14 weeks of age of experimental protocol B. The data shown are of the Control (males *n* = 9, females *n* = 9), C+10% 7w (males *n* = 6, females *n* = 6), C+10% 11w (males *n* = 6, females *n* = 6), C+30% 7w (males *n* = 6, females *n* = 6), C+30% 11w (males *n* = 6, females *n* = 7), Stz70 (males *n* = 8, females *n* = 9), Stz70+10% 7w (males *n* = 7, females *n* = 6), Stz70+10% 11w (males *n* = 6, females *n* = 5), Stz70+30% 7w (males *n* = 6, females *n* = 6), and Stz70+30% 11w (males *n* = 6, females *n* = 6) groups. Values are expressed as the median and interquartile range (IQR) (p25-p75). Variation between more than two groups was measured by the Kruskal-Wallis test followed by the Dunn Multiple Comparison Test. The asterisk denotes that the data are significantly different from the Control group (^∗^*p* < 0.05, ^∗∗^*p* < 0.01, and ^∗∗∗^*p* < 0.001), and the plus sign denotes that the data are significantly different from the Stz70 group (^+^*p* < 0.05, ^++^*p* < 0.01, and ^+++^*p* < 0.001).

	Male		Female	
	10^th^ week	14^th^ week	10^th^ week	14^th^ week
Control	97 (91.25-116.8)	106.5 (94.5-119.5)	93 (92-118)	96.5 (89.5-105)
C+10% 7w	110 (91-121)	111 (98.5-130.5)	103 (99-107.5)	113 (104.8-118.5)
C+10% 11w	110 (99-161)	124 (107.3-131.8)	105.5 (98-118)	109.5 (100.3-114.3)
C+30% 7w	99.5 (94.7-108)	92.5 (84.7-109)	105 (94.5-111)	100 (94.2-104.3)
C+30% 11w	111.3 (98.7-129.8)	104 (94-111.8)	117 (98.5-125)	115 (94.7-124.5)
Stz70	129.5 (120.3-144.8)	131 (107-248)	131.5 (106.5-153.5)	138.5∗∗ (113-206)
Stz70+10% 7w	125 (99.7-165.3)	**277**∗ (150.8-361.3)	145.5 (138.5151)	**190**∗∗∗ (159.3-223.3)
Stz70+10% 11w	137.5 (113-186.3)	**186.5**∗ (171.8-295.3)	125.5 (122-129)	**172.5**∗∗ (139-206)
Stz70+30% 7w	119.5 (99.7-126.5)	157 (136-226.8)	138 (116.5155)	**160.5**∗∗∗ (140.8-183.8)
Stz70+30% 11w	108 (99-131)	154 (120.3-208.5)	110 (83-125.5)	**173.8**∗∗∗ (156.8-202.4)

**Table 4:** Glucose tolerance test (GTT) and insulin tolerance test (ITT) of rats of experimental protocol B. The data shown are of the Control (males *n* = 9, females *n* = 9), C+10% 7w (males *n* = 6, females *n* = 6), C+10% 11w (males *n* = 6, females *n* = 6), C+30% 7w (males *n* = 6, females *n* = 6), C+30% 11w (males *n* = 6, females *n* = 7), Stz70 (males *n* = 8, females *n* = 9), Stz70+10% 7w (males *n* = 7, females *n* = 6), Stz70+10% 11w (males *n* = 6, females *n* = 5), Stz70+30% 7w (males *n* = 6, females *n* = 6), and Stz70+30% 11w (males *n* = 6, females *n* = 6) groups. Data are represented in percentages, considering the initial glucose value as 100%. Values (except initial glucose) are expressed as the median and interquartile range (IQR) (p25-p75). For the GTT, the rats were given glucose (2 g/kg body weight) orally, and for the ITT, they were injected with insulin (0.5 IU/kg body weight) i.p. Blood glucose level was monitored at 30, 60, and 120 min after the administration of glucose or insulin. The area under the curve (AUC) was calculated using the trapezoidal rule in GraphPad Prism. Variation between more than two groups was measured by the Kruskal-Wallis test followed by the Dunn Multiple Comparison Test. The asterisk denotes that the data are significantly different from the Control group (^∗^*p* < 0.05, ^∗∗^*p* < 0.01, and ^∗∗∗^*p* < 0.001), and the plus sign denotes that the data are significantly different from the Stz70 group (^+^*p* < 0.05, ^++^*p* < 0.01, and ^+++^*p* < 0.001).

		GTT					ITT				
		0 min	30 min	60 min	120 min	AUC	0 min	30 min	60 min	120 min	AUC
Male	Control	100	147.5 (116.1-180.6)	133.1 (129.7-156)	114.3 (97.46-133)	505.4 (459.4-575.2)	100	55.4 (51.08-63.73)	68.8 (48.96-74.9)	80 (58.6-89.3)	288.7 (247.2-303.2)
	C+10% 7w	100	151.3 (118.5-167.7)	137.7 (104.6-178.6)	98.8 (93.86-119.5)	532.3 (429.2-569.4)	100	**82.9**∗ (62.6-87.8)	62.22 (55.1-88.2)	80.18 (76.8-123.5)	334 (274.2-377.8)
	C+10% 11w	100	144.3 (123.8-154.1)	131.2 (105.4-146.2)	104.9 (77.34-129.6)	474.4 (429.4-543.6)	100	69.5∗ (63.2-73)	79.1 (71.7-88.3)	95.2 (91-104.3)	**341.4**∗∗**(320.8-346.7)**
	C+30% 7w	100	202.4 (165.5-235.5)	175.4∗ (149.7-202.9)	153.9∗ (126.4-177.2)	650.4∗ (573-775.4)	100	**85.91**∗∗ (69.4-100.3)	92.94 (65-97.6)	100∗ (87.1-117)	**375.4**∗**(298.2-426.8)**
	C+30% 11w	100	176.2 (146.1-203)	143.4 (132.4-168)	129.5 (115.8-156.3)	596.8 (521.8-640.8)	100	72.9∗ (60.3-84.3)	76.6 (50.5-92.6)	104.8∗ (81.3-132.3)	343.5 (290-378.1)
	Stz70	100	236∗∗ (190.9-279.4)	216.7∗∗ (195.5-247.7)	184.1∗∗ (151.3-192.2)	796.2∗∗∗ (689.5-860.8)	100	**63.57**∗ (61.8-89.8)	58.02 (37.1-66.7)	76.58 (48.2-98.8)	291.4 (268.2-369.1)
	Stz70+10% 7w	100	205.3 (162.2-232.5)	205.7∗ (159.3-230.1)	158.2 (114.8-184.9)	722.1∗ (568.9-809.7)	100	**86.49**∗ (58.3-115.2)	55.56 (24.1-76)	49.69 (23-89.5)	289.3 (165.4-364.1)
	Stz70+10% 11w	100	209.8 (145.5-280.9)	202.7 (142.3-219.6)	156.3 (108.8-188.8)	737.8 (526.7-822.4)	100	72.58 (48.9-91.8)	56.56 (33.4-81.1)	72.59 (41.3-88.8)	291.5 (191.2-339.8)
	Stz70+30% 7w	100	282.2 (172.1-303.8)	278.3∗ (172.4-322.3)	228.3∗ (143.1-242)	984.6∗ (633-1064)	100	65.09 (52.6-69.2)	40.57 (37.4-78.1)	40.54 (34.6-66.8)	215.4 (193.8-314.7)
	Stz70+30% 11w	100	242.7∗ (208.9-270.8)	254.5∗ (160.7-291.4)	202.3 (126.9-224.4)	876.8∗ (626.9-982.4)	100	55.9^+^ (52.7-65)	37.3 (34.6-55.4)	57.8 (51.4-92.3)	229.8 (208.3-283)

Female	Control	100	174.7 (143.9-196.4)	149 (125.3-160.5)	113.3 (101.5-149.7)	547.5 (484.1-632.7)	100	54.4 (52.3-60.2)	64.2 (48.5-75)	87.5 (72.7-101.1)	315.9 (258.2-352)
	C+10% 7w	100	151 (125.6-171.3)	142.3 (122.6-156.7)	107.6 (90.11-138.9)	513.8 (487.6-565.6)	100	57.58 (55.4-59.6)	52.08 (49.9-54.4)	81.3 (67-93.5)	265.6 (258.1-275.2)
	C+10% 11w	100	169 (150-216.3)	132.1 (120.4-152.1)	113.1 (101.7-142.9)	509 (492.3-637.3)	100	60.8 (52.2-67.2)	59.45 (42.5-72)	86.6 (73.6-110)	286.8 (243.6-330.7)
	C+30% 7w	100	145.2 (125.7-166.6)	161.8 (128.6-200.7)	113.8 (103.2-133.1)	563.9 (417.7-638.6)	100	56.76 (51.9-67.8)	47.8 (39.7-61.5)	64.1∗ (56.3-82.7)	248.4 (217.7-286.9)
	C+30% 11w	100	217 (125.7-236.5)	149 (116.9-177.5)	126.4 (102.9-158.6)	631.4 (427.7-708.5)	100	57.83 (51.9-90.5)	62.3 (56.1-87.8)	101.1 (85.2-161.9)	301.9 (263.3-437.4)
	Stz70	100	253.7∗∗ (205.7-286.5)	243.4∗∗∗ (227.8-277.2)	158.3 (118.4-220.6)	822.4∗∗ (803.7-926.2)	100	73.3 (56.9-123.5)	75.2 (49.6-121.7)	110.5 (79.8-142.9)	318.8 (230.6-342.9)
	Stz70+10% 7w	100	198 (178-272.4)	175.6∗ (167.5-189.7)	82.7∗∗^+^ (71.03-90.24)	630 (582-677.9)	100	37.6∗∗^++^ (34.7-40.4)	32.8∗∗^+^ (30.1-35.6)	47.6∗∗^+^ (47-48.2)	184.4∗∗^++^ (182.5-186.2)
	Stz70+10% 11w	100	225.7∗ (225.7-282.4)	196.1∗∗ (196.1-243.4)	138.3^+^ (125-138.3)	708.3∗ (693-725.2)	100	63.53 (60.2-66.9)	49.63 (49.6-49.6)	82.05 (79.1-85)	270.5 (221.3-299)
	Stz70+30% 7w	100	260.5∗∗∗ (251.1-275.4)	259.1∗∗∗ (219.5-284)	145.5 (108.7-182.9)	859∗∗∗ (752.5-903.7)	100	45.14 (39.5-65.5)	41.08∗ (31.1-55.7)	49.69∗ (43.7-80.5)	199.6∗^++^ (185.9-286.4)
	Stz70+30% 11w	100	252.5∗ (205.7-282.9)	286.6∗∗ (268.8-329.7)	238.2∗∗ (220.6-253.8)	1081∗∗ (994-1245)	100	51.34 (41.9-60.8)	59.95 (56.2-63.7)	86.06 (85-87.2)	277.4 (274.6-280.1)

**Table 5:** Body weight gain (g in body weight) and food intake daily (g of food/100 g of body weight). The data shown are of the rats of experimental protocols A and B. Body weight and food intake were monitored daily for 6 weeks (8-4 weeks of age). Values are expressed as the median and interquartile range (IQR) (p25-p75). Variation between more than two groups was measured by the Kruskal-Wallis test followed by the Dunn Multiple Comparison Test. The asterisk denotes that the data are significantly different from the Control group (^∗^*p* < 0.05, ^∗∗^*p* < 0.01, and ^∗∗∗^*p* < 0.001), and the plus sign denotes that the data are significantly different from the Stz70 group (^+^*p* < 0.05, ^++^*p* < 0.01, and ^+++^*p* < 0.001).

	Body weight gain		Food intake daily	
	Male	Female	Male	Female
Control	152.6 (138.7-170)	78.2 (66.1-85)	6.9 (6.5-7.4)	7.7 (7.1-8.4)
Stz70	150.5 (122.2-166.4)	68.1 (56.3-77.7)	7.6 (6.8-8.9)	8 (7.3-10.3)
Stz90	**99.9**∗∗^**+**^ (74.3-121.5)	51∗ (36.1-65.9)	12.2∗∗∗^+++^ (11.3-12.9)	**13.2**∗∗∗^++^ (11.8-14.1)
C+10% 7w	178.6 (137.8-190)	62.6 (62-77.8)	6.9 (6.5-7.1)	7.2 (6.4-7.6)
C+10% 11w	**182.6**∗∗ (179-201.8)	93.3 (79.5-95.1)	4.6∗∗∗ (4.1-4.9)	4.5∗∗∗ (3.8-5)
C+30% 7w	**177**∗ (160.5-202.8)	66.6 (50.5-74.1)	6.9 (6.1-7.6)	7.7 (5.9-8.8)
C+30% 11w	165.7 (128.9-185.3)	83.1 (70.8-113.6)	4.5∗∗∗ (4-4.7)	3.8∗∗∗ (3.3-5.7)
Stz70+10% 7w	**106.8**∗∗ (90.6-131.7)	72.3 (62.6-108.7)	**12.3**∗∗∗^+++^ (11.3-13.5)	7.8 (6.5-9.3)
Stz70+10% 11w	**89.9**∗∗∗^++^ (56.6-97.9)	88.8 (78.2-105.3)	4.6∗∗^++^ (4.1-5.4)	5.5∗∗∗^+++^ (4.4-5.6)
Stz70+30% 7w	145.4 (141.8-150)	67.4 (66.9-87.3)	9.5∗ (6.6-10.7)	7.3+ (6.7-8)
Stz70+30% 11w	130.9 (102-170)	83.6+ (74.4-92.9)	4.3∗∗∗^+++^ (3.8-4.4)	4.2∗∗∗^+++^ (3.8-4.9)

## Data Availability

The data used to support the findings of this study are included within the article.
